# A regressed germ cell tumor discovered secondary to azoospermia

**DOI:** 10.1002/iju5.12552

**Published:** 2022-11-08

**Authors:** Hiroki Tsujioka, Keiichiro Uemura, Toshiyuki Iwahata, Yasuyuki Inoue, Minoru Inoue, Akiyoshi Osaka, Akinori Nakayama, Hiroshi Okada, Kazutaka Saito

**Affiliations:** ^1^ Department of Urology Dokkyo Medical University Saitama Medical Center Koshigaya Saitama Japan; ^2^ Department of Urology Kurume University Kurume Fukuoka Japan

**Keywords:** azoospermia, regressed germ cell tumor, seminoma

## Abstract

**Introduction:**

Regressed germ cell tumors are a rare disease commonly diagnosed with metastatic symptoms without local symptoms in the testis.

**Case presentation:**

A 33‐year‐old man with azoospermia was referred to our hospital. His right testis was slightly swollen, and ultrasonography revealed hypoechogenicity of the right testis with decreased blood flow. Right high orchiectomy was performed. Pathologically, the seminiferous tubules were absent or highly atrophied with vitrification degeneration; however, no neoplastic lesion was confirmed. One‐month post‐surgery, the patient noticed a mass in the left supraclavicular fossa, of which a biopsy revealed seminoma. The patient was diagnosed with a regressed germ cell tumor and underwent systemic chemotherapy.

**Conclusion:**

We reported the first case of a regressed germ cell tumor discovered due to complaints of azoospermia.

**Abbreviations & Acronyms**
AFP: alpha‐fetoprotein
CT: computed tomography
HCG: human chorionic gonadotrophin
LDH: lactate dehydrogenase
MD‐TESE: microdissection testicular sperm extraction
MRI: magnetic resonance imaging

**Keynote message**

When examining a patient with complaints of azoospermia, palpation and ultrasonography should be performed to explore possible testicular tumors.

## Introduction

Regressed germ cell tumors are a rare disease in which only necrosis, scar tissue, or regressed teratomas are found in the testes of patients with confirmed extragonadal germ cell tumors.^1^ Local symptoms in the testis are uncommon, and tumors typically go undetected until metastatic lesions progress. Although testicular cancer has been reported to be associated with infertility, to our knowledge, regressed germ cell tumors found due to azoospermia have not been reported. Herein, we report the case of a regressed germ cell tumor with a chief complaint of azoospermia.

## Case report

**Fig. 1:**
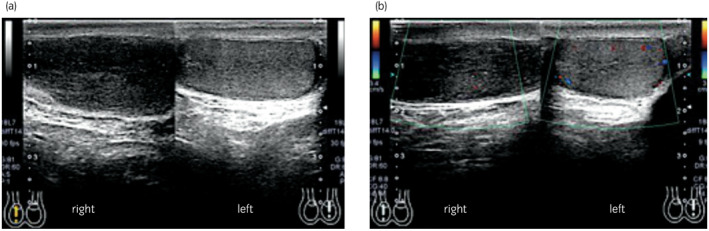
Ultrasonography indicates a homogeneous internal hypoechoic lesion in the right testis (a). Color doppler ultrasonography reveals decreased blood flow in the lesion (b).

**Fig. 2:**
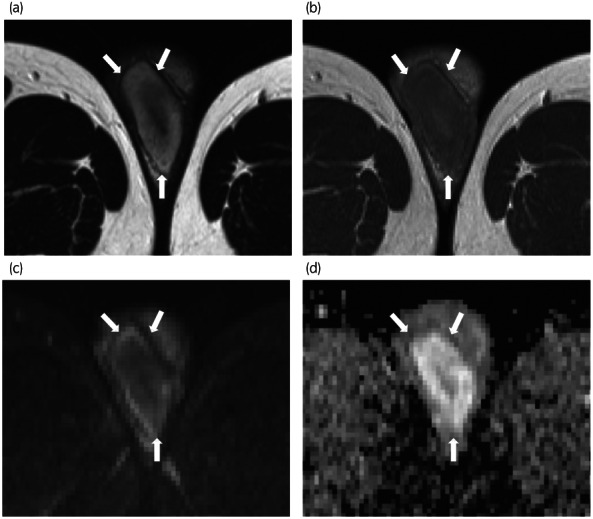
MRI indicating a hypointense lesion at T2 in the center of the right testis (a), and poor contrast effects compared to the contralateral side (b). Diffusion‐weighted imaging confirming hypointensity at the same site (c), and an apparent diffusion coefficient indicating hyperintensity (d).

**Fig. 3:**
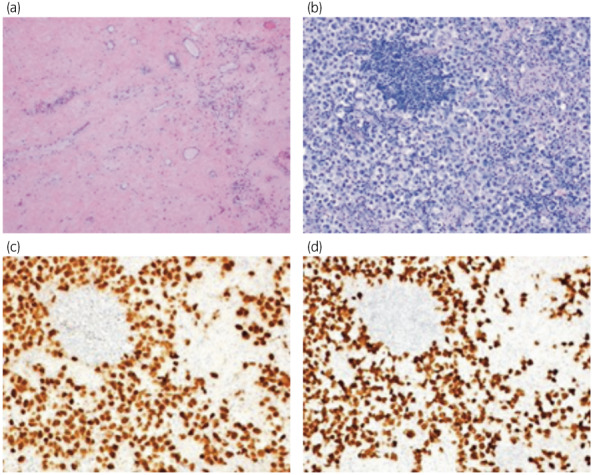
Microscopic examination using hematoxylin and eosin staining demonstrating scarring and extensive fibrosis in the testicular parenchyma (a) (objective 100×), and large round tumor cells with clear round nuclei of nucleoli and a clear cytoplasm in a cervical lymph node (b) (objective 400×). Immunohistochemical examinations indicates that the tumor cells are OCT3/4 positive (c) and SALL4 positive (d) (objective 400×).

A 33‐year‐old man visited another hospital due to infertility and was identified by semen analysis as azoospermia, so he was referred to our hospital. The right testis was slightly swollen, with elastic hardness. Ultrasonography revealed a homogeneous internal hypoechoic lesion with decreased blood flow (Fig. 1a,d). MRI showed a hypointense lesion on T2‐weighted imaging and diffusion‐weighted imaging, and poor contrast effects, suggesting testicular infarction and ischemic necrosis (Fig. 2a–d). CT revealed mild swelling of the para‐aortic lymph nodes that were within the physiological range. Tumor markers, including LDH, AFP, HCG, and soluble interleukin −2 receptor, were all negative. Malignancy could not be ruled out in the right testis, so right high orchiectomy and left microdissection testicular sperm extraction (MD‐TESE) were performed. Macroscopically, the right testis was occupied by a homogeneous white‐toned mass, and the typical testicular structure, including the seminiferous tubules, was not identified. Histopathological examination of the right testis revealed an absence of most of the seminiferous tubules, with the remaining tubules being highly atrophied. Furthermore, the testicular tissue showed vitrification degeneration and sparse infiltration of inflammatory cells (Fig. 3a). MD‐TESE in the left testis failed to retrieve sperm. Histopathological analysis of the left testis revealed an absence of germ cells, with only Sertoli cells present. No neoplastic lesions were identified in the excised tissue.

One month post‐surgery, the patient noticed a mass on the left side of his neck. A hard mass with poor mobility was palpated in the left supraclavicular fossa. Tumor markers, including LDH, AFP, and HCG, were all negative. CT confirmed a 37 × 21 mm mass in the lymph nodes of the left supraclavicular fossa and a 22 × 19 mm mass in the lymph nodes of the para‐aorta. A cervical lymph node biopsy was performed, and histopathology revealed large round tumor cells with clear, round nuclei of nucleoli and clear cytoplasm. The tumor cells were OCT3/4 and SALL positive (Fig. 3b–d). Therefore, the cervical lymph node tissue was diagnosed as metastatic seminoma. The patient was diagnosed with a regressed germ cell tumor. Systemic chemotherapy (Bleomycin, Etoposide, Cisplatin) was initiated, and a total of three courses were implemented. During chemotherapy, tumor markers, including LDH, AFP, and HCG, were all negative. After systemic chemotherapy, the swollen lymph nodes shrank, and the patient had no recurrence 13 months after chemotherapy.

## Discussion

To our knowledge, this is the first report on a regressed germ cell tumor discovered due to complaints of azoospermia. Regressed germ cell tumors, testicular germ cell tumors presenting with spontaneous complete or partial regression, are commonly diagnosed with metastatic symptoms, such as extragonadal masses without testicular symptoms. Less than 5% of testicular germ cell tumors spontaneously regress; thus, regressed germ cell tumors are rare.^2^ Commonly, necrosis, scar tissue, or regressed teratomas may be found in the testes. Although the pathophysiology has not been established, two possible mechanisms have been proposed: ischemic regression caused by ischemia of the tumor itself and immune‐modulated regression due to immune system disorders.^3^

In patients with regressive germ cell tumors, the main initial symptoms are abdominal pain (32.8%), lower back pain (28.1%), and palpation of tumors in the abdomen, neck, and supraclavicular region (20.3%).^3^ In contrast, only 7.8% of patients complained of local testicular symptoms, including swelling of the scrotum, palpation of a mass, and pain, as initial symptoms.^3^ In this case, a regressed germ cell tumor was incidentally found during the assessment for azoospermia. Therefore, this case report is interesting and significant because it demonstrates the natural history of a regressed germ cell tumor diagnosed at a relatively early stage, followed by the detection of metastasis.

The correlation between infertility with testicular cancer has been shown. Patients with testicular cancer often have poor semen quality, with semen abnormalities identified in approximately 50% of patients.^4^ Interestingly, infertile men with semen abnormalities are 20 times more likely to develop testicular cancer.^5^ The underlying mechanisms of declining semen quality in testicular cancer could be due to mechanical loss of physical testicular volume in the affected testis, paracrine and endocrine effects on the ipsilateral and contralateral testis from the tumor, and congenital factors.^6^ In this case, no germ cells were identified in the left testis and the right affected testis. This could be due to paracrine or endocrine effects from the tumor before regression on bilateral testes or congenital factors. Therefore, palpation and ultrasonography should be performed when examining a patient with abnormal semen to explore possible testicular tumors.

## Conclusions

We reported a patient with a regressed germ cell tumor following semen and testicular structure analysis for azoospermia. This report could increase awareness on the consideration of possible regressed germ cell tumors in patients with severe semen abnormalities and abnormal masses or tissue in the testes.

## Author contributions

Hiroki Tsujioka: Writing – original draft. Keiichiro Uemura: Conceptualization; project administration. Toshiyuki Iwahata: Conceptualization. Yasuyuki Inoue: Conceptualization. Minoru Inoue: Conceptualization. Akiyoshi Osaka: Conceptualization. Akinori Nakayama: Conceptualization. Hiroshi Okada: Supervision. Kazutaka Saito: Supervision.

## Conflict of interest

The authors declare no conflict of interest.

## Approval of the research protocol by an Institutional Reviewer Board

This study protocol was reviewed and approved by Dokkyo Medical University Saitama Medical Center Ethical Committee (approval number: 22006).

## Informed consent

Written informed consent was obtained from all patients.

## Registry and the Registration No. of the study/trial

Not applicable.
